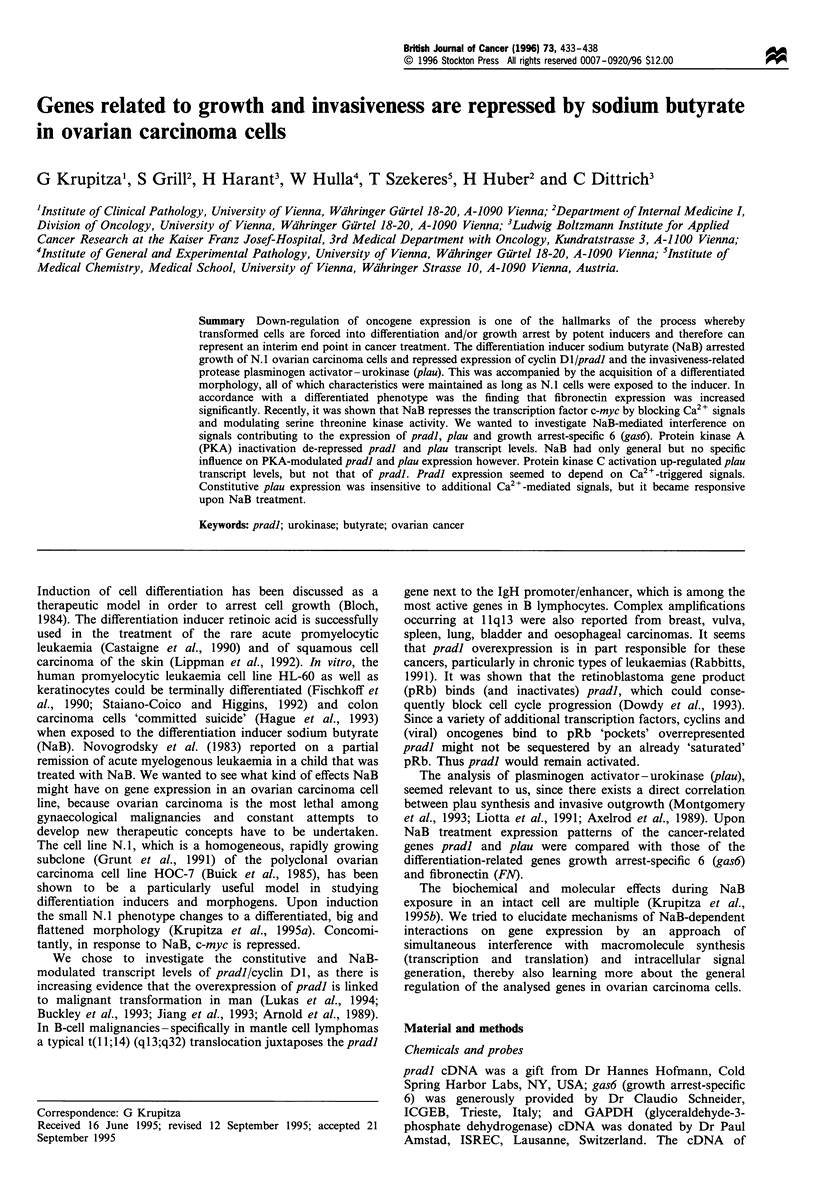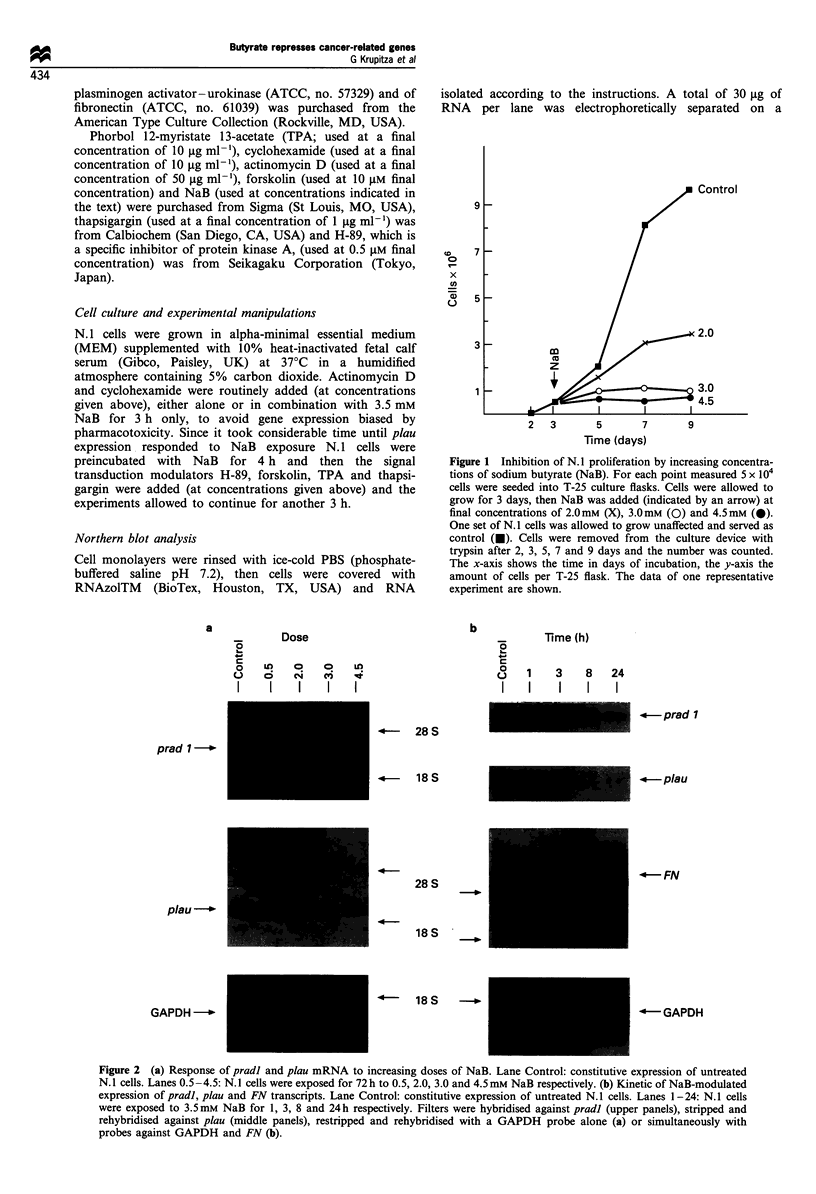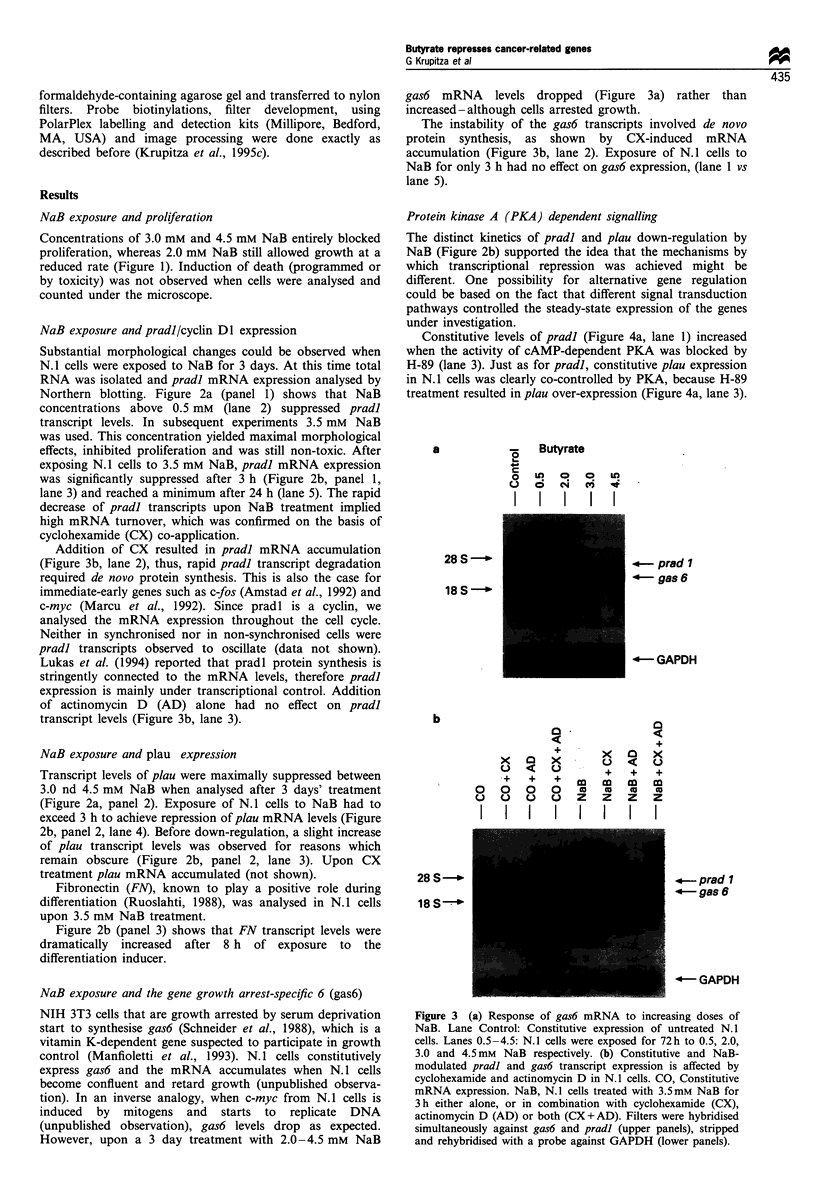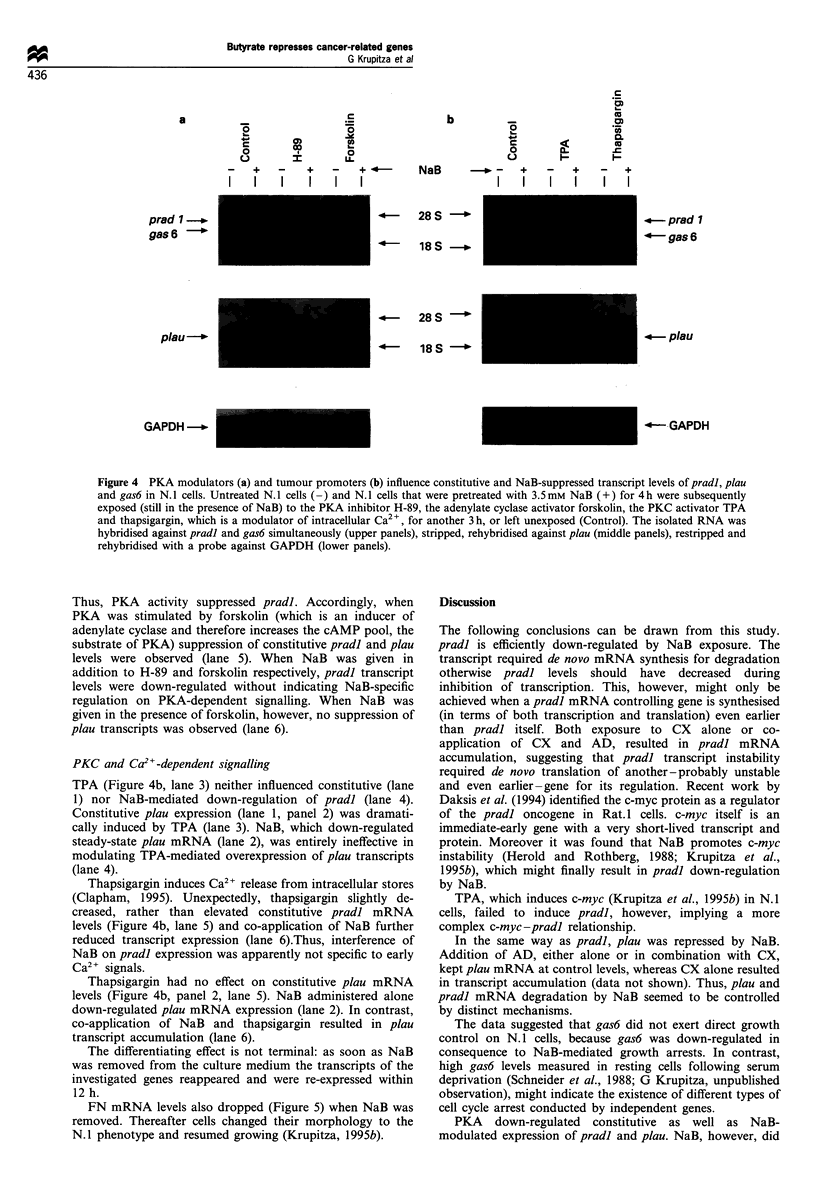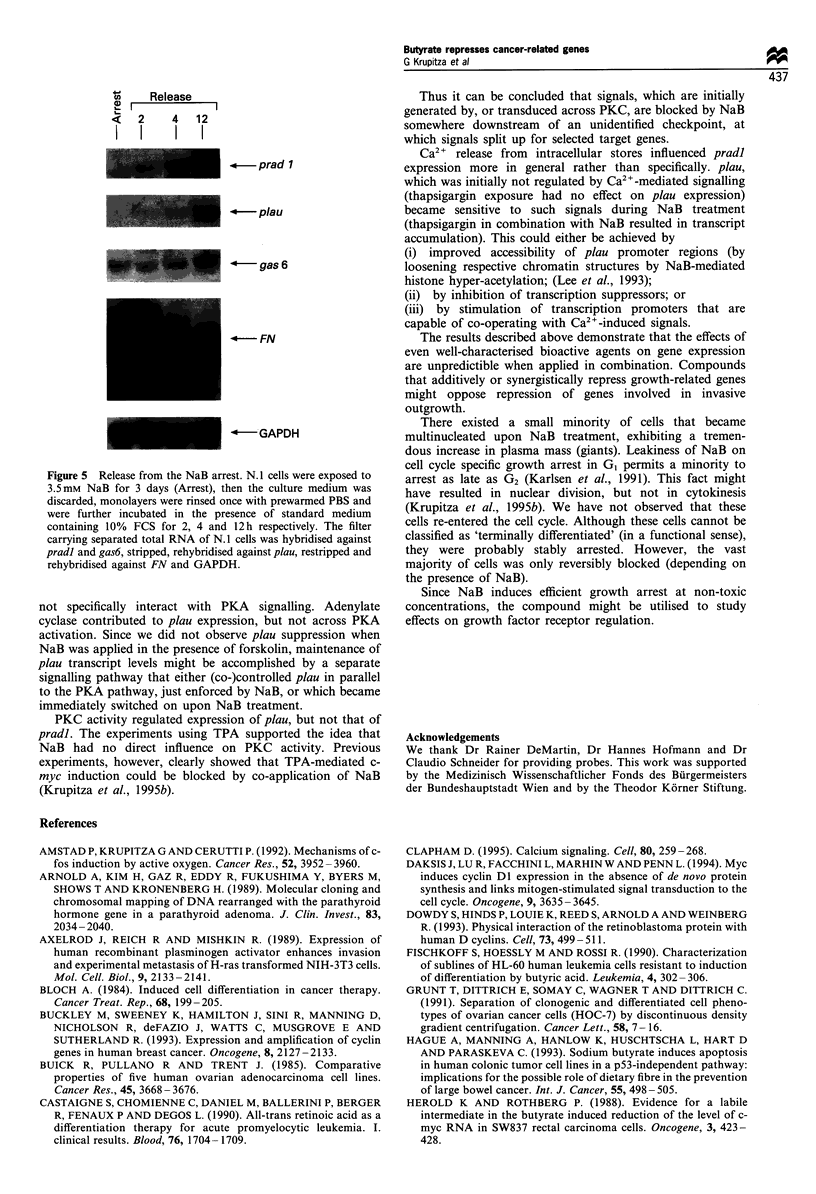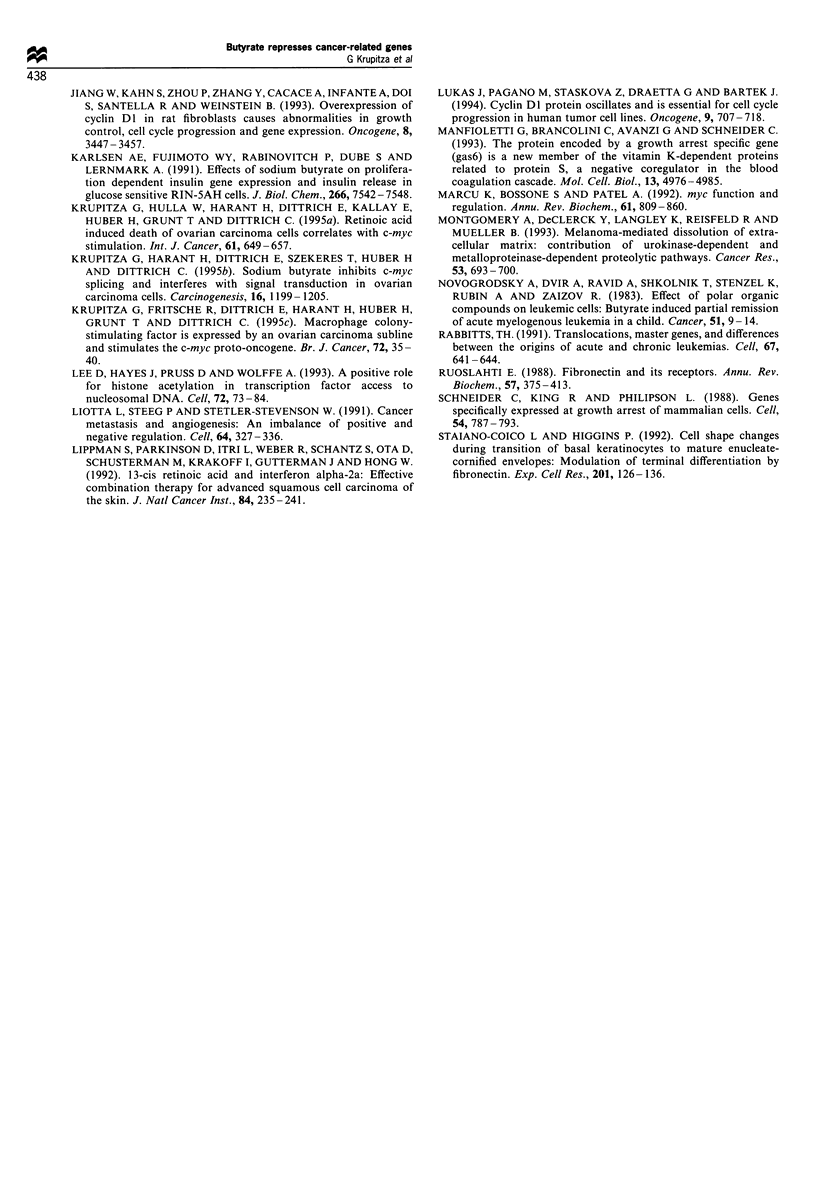# Genes related to growth and invasiveness are repressed by sodium butyrate in ovarian carcinoma cells.

**DOI:** 10.1038/bjc.1996.78

**Published:** 1996-02

**Authors:** G. Krupitza, S. Grill, H. Harant, W. Hulla, T. Szekeres, H. Huber, C. Dittrich

**Affiliations:** Institute of Clinical Pathology, University of Vienna, Austria.

## Abstract

**Images:**


British Journal of Cancer (1996) 73, 433-438
© 1996 Stockton Press All rights reserved 0007-0920/96 $12.00

### Genes related to growth and invasiveness are repressed by sodium butyrate in ovarian carcinoma cells

G Krupitza[1], S Grill[2], H Harant[3], W Hulla[4], T Szekeres[5], H Huber[2] and C Dittrich[3]

[1]*Institute of Clinical Pathology, University of Vienna, Währinger Gürtel 18-20, A-1090 Vienna;* [2]*Department of Internal Medicine I, Division of Oncology, University of Vienna, Währinger Gürtel 18-20, A-1090 Vienna;* [3]*Ludwig Boltzmann Institute for Applied Cancer Research at the Kaiser Franz Josef-Hospital, 3rd Medical Department with Oncology, Kundratstrasse 3, A-1100 Vienna;* [4]*Institute of General and Experimental Pathology, University of Vienna, Währinger Gürtel 18-20, A-1090 Vienna;* [5]*Institute of Medical Chemistry, Medical School, University of Vienna, Währinger Strasse 10, A-1090 Vienna, Austria.*

**Summary** Down-regulation of oncogene expression is one of the hallmarks of the process whereby transformed cells are forced into differentiation and/or growth arrest by potent inducers and therefore can represent an interim end point in cancer treatment. The differentiation inducer sodium butyrate (NaB) arrested growth of N.1 ovarian carcinoma cells and repressed expression of cyclin D1/*pradl* and the invasiveness-related protease plasminogen activator-urokinase (*plau*). This was accompanied by the acquisition of a differentiated morphology, all of which characteristics were maintained as long as N.1 cells were exposed to the inducer. In accordance with a differentiated phenotype was the finding that fibronectin expression was increased significantly. Recently, it was shown that NaB represses the transcription factor c-*myc* by blocking $Ca^{2+}$ signals and modulating serine threonine kinase activity. We wanted to investigate NaB-mediated interference on signals contributing to the expression of *pradl*, *plau* and growth arrest-specific 6 (*gas6*). Protein kinase A (PKA) inactivation de-repressed *pradl* and *plau* transcript levels. NaB had only general but no specific influence on PKA-modulated *pradl* and *plau* expression however. Protein kinase C activation up-regulated *plau* transcript levels, but not that of *pradl*. *Pradl* expression seemed to depend on $Ca^{2+}$-triggered signals. Constitutive *plau* expression was insensitive to additional $Ca^{2+}$-mediated signals, but it became responsive upon NaB treatment.

**Keywords:** *pradl*; urokinase; butyrate; ovarian cancer

Induction of cell differentiation has been discussed as a therapeutic model in order to arrest cell growth (Bloch, 1984). The differentiation inducer retinoic acid is successfully used in the treatment of the rare acute promyelocytic leukaemia (Castaigne *et al.*, 1990) and of squamous cell carcinoma of the skin (Lippman *et al.*, 1992). *In vitro*, the human promyelocytic leukaemia cell line HL-60 as well as keratinocytes could be terminally differentiated (Fischkoff *et al.*, 1990; Staiano-Coico and Higgins, 1992) and colon carcinoma cells 'committed suicide' (Hague *et al.*, 1993) when exposed to the differentiation inducer sodium butyrate (NaB). Novogrodsky *et al.* (1983) reported on a partial remission of acute myelogenous leukaemia in a child that was treated with NaB. We wanted to see what kind of effects NaB might have on gene expression in an ovarian carcinoma cell line, because ovarian carcinoma is the most lethal among gynaecological malignancies and constant attempts to develop new therapeutic concepts have to be undertaken. The cell line N.1, which is a homogeneous, rapidly growing subclone (Grunt *et al.*, 1991) of the polyclonal ovarian carcinoma cell line HOC-7 (Buick *et al.*, 1985), has been shown to be a particularly useful model in studying differentiation inducers and morphogens. Upon induction the small N.1 phenotype changes to a differentiated, big and flattened morphology (Krupitza *et al.*, 1995*a*). Concomitantly, in response to NaB, c-*myc* is repressed.

We chose to investigate the constitutive and NaB-modulated transcript levels of *pradl*/cyclin D1, as there is increasing evidence that the overexpression of *pradl* is linked to malignant transformation in man (Lukas *et al.*, 1994; Buckley *et al.*, 1993; Jiang *et al.*, 1993; Arnold *et al.*, 1989). In B-cell malignancies-specifically in mantle cell lymphomas a typical t(11;14) (q13;q32) translocation juxtaposes the *pradl* gene next to the IgH promoter/enhancer, which is among the most active genes in B lymphocytes. Complex amplifications occurring at 11q13 were also reported from breast, vulva, spleen, lung, bladder and oesophageal carcinomas. It seems that *pradl* overexpression is in part responsible for these cancers, particularly in chronic types of leukaemias (Rabbitts, 1991). It was shown that the retinoblastoma gene product (pRb) binds (and inactivates) *pradl*, which could consequently block cell cycle progression (Dowdy *et al.*, 1993). Since a variety of additional transcription factors, cyclins and (viral) oncogenes bind to pRb 'pockets' overrepresented *pradl* might not be sequestered by an already 'saturated' pRb. Thus *pradl* would remain activated.

The analysis of plasminogen activator-urokinase (*plau*), seemed relevant to us, since there exists a direct correlation between plau synthesis and invasive outgrowth (Montgomery *et al.*, 1993; Liotta *et al.*, 1991; Axelrod *et al.*, 1989). Upon NaB treatment expression patterns of the cancer-related genes *pradl* and *plau* were compared with those of the differentiation-related genes growth arrest-specific 6 (*gas6*) and fibronectin (*FN*).

The biochemical and molecular effects during NaB exposure in an intact cell are multiple (Krupitza *et al.*, 1995*b*). We tried to elucidate mechanisms of NaB-dependent interactions on gene expression by an approach of simultaneous interference with macromolecule synthesis (transcription and translation) and intracellular signal generation, thereby also learning more about the general regulation of the analysed genes in ovarian carcinoma cells.

### Material and methods

### *Chemicals and probes*

*pradl* cDNA was a gift from Dr Hannes Hofmann, Cold Spring Harbor Labs, NY, USA; *gas6* (growth arrest-specific 6) was generously provided by Dr Claudio Schneider, ICGEB, Trieste, Italy; and GAPDH (glyceraldehyde-3-phosphate dehydrogenase) cDNA was donated by Dr Paul Amstad, ISREC, Lausanne, Switzerland. The cDNA of

Correspondence: G Krupitza
Received 16 June 1995; revised 12 September 1995; accepted 21 September 1995

plasminogen activator–urokinase (ATCC, no. 57329) and of fibronectin (ATCC, no. 61039) was purchased from the American Type Culture Collection (Rockville, MD, USA).

Phorbol 12-myristate 13-acetate (TPA; used at a final concentration of 10 μg $ml^{-1}$), cyclohexamide (used at a final concentration of 10 μg $ml^{-1}$), actinomycin D (used at a final concentration of 50 μg $ml^{-1}$), forskolin (used at 10 μM final concentration) and NaB (used at concentrations indicated in the text) were purchased from Sigma (St Louis, MO, USA), thapsigargin (used at a final concentration of 1 μg $ml^{-1}$) was from Calbiochem (San Diego, CA, USA) and H-89, which is a specific inhibitor of protein kinase A, (used at 0.5 μM final concentration) was from Seikagaku Corporation (Tokyo, Japan).

*Cell culture and experimental manipulations*

N.1 cells were grown in alpha-minimal essential medium (MEM) supplemented with 10% heat-inactivated fetal calf serum (Gibco, Paisley, UK) at 37°C in a humidified atmosphere containing 5% carbon dioxide. Actinomycin D and cyclohexamide were routinely added (at concentrations given above), either alone or in combination with 3.5 mM NaB for 3 h only, to avoid gene expression biased by pharmacotoxicity. Since it took considerable time until *plau* expression responded to NaB exposure N.1 cells were preincubated with NaB for 4 h and then the signal transduction modulators H-89, forskolin, TPA and thapsigargin were added (at concentrations given above) and the experiments allowed to continue for another 3 h.

*Northern blot analysis*

Cell monolayers were rinsed with ice-cold PBS (phosphate-buffered saline pH 7.2), then cells were covered with RNAzolTM (BioTex, Houston, TX, USA) and RNA isolated according to the instructions. A total of 30 μg of RNA per lane was electrophoretically separated on a

**Figure 1** Inhibition of N.1 proliferation by increasing concentrations of sodium butyrate (NaB). For each point measured $5 \times 10^4$ cells were seeded into T-25 culture flasks. Cells were allowed to grow for 3 days, then NaB was added (indicated by an arrow) at final concentrations of 2.0 mM (X), 3.0 mM (○) and 4.5 mM (●). One set of N.1 cells was allowed to grow unaffected and served as control (■). Cells were removed from the culture device with trypsin after 2, 3, 5, 7 and 9 days and the number was counted. The *x*-axis shows the time in days of incubation, the *y*-axis the amount of cells per T-25 flask. The data of one representative experiment are shown.

**Figure 2** (**a**) Response of *pradl* and *plau* mRNA to increasing doses of NaB. Lane Control: constitutive expression of untreated N.1 cells. Lanes 0.5–4.5: N.1 cells were exposed for 72 h to 0.5, 2.0, 3.0 and 4.5 mM NaB respectively. (**b**) Kinetic of NaB-modulated expression of *pradl*, *plau* and *FN* transcripts. Lane Control: constitutive expression of untreated N.1 cells. Lanes 1–24: N.1 cells were exposed to 3.5 mM NaB for 1, 3, 8 and 24 h respectively. Filters were hybridised against *pradl* (upper panels), stripped and rehybridised against *plau* (middle panels), restripped and rehybridised with a GAPDH probe alone (**a**) or simultaneously with probes against GAPDH and *FN* (**b**).

formaldehyde-containing agarose gel and transferred to nylon filters. Probe biotinylations, filter development, using PolarPlex labelling and detection kits (Millipore, Bedford, MA, USA) and image processing were done exactly as described before (Krupitza *et al.*, 1995*c*).

### Results

### *NaB exposure and proliferation*

Concentrations of 3.0 mM and 4.5 mM NaB entirely blocked proliferation, whereas 2.0 mM NaB still allowed growth at a reduced rate (Figure 1). Induction of death (programmed or by toxicity) was not observed when cells were analysed and counted under the microscope.

### *NaB exposure and pradl*/cyclin D1 expression

Substantial morphological changes could be observed when N.1 cells were exposed to NaB for 3 days. At this time total RNA was isolated and *pradl* mRNA expression analysed by Northern blotting. Figure 2a (panel 1) shows that NaB concentrations above 0.5 mM (lane 2) suppressed *pradl* transcript levels. In subsequent experiments 3.5 mM NaB was used. This concentration yielded maximal morphological effects, inhibited proliferation and was still non-toxic. After exposing N.1 cells to 3.5 mM NaB, *pradl* mRNA expression was significantly suppressed after 3 h (Figure 2b, panel 1, lane 3) and reached a minimum after 24 h (lane 5). The rapid decrease of *pradl* transcripts upon NaB treatment implied high mRNA turnover, which was confirmed on the basis of cyclohexamide (CX) co-application.

Addition of CX resulted in *pradl* mRNA accumulation (Figure 3b, lane 2), thus, rapid *pradl* transcript degradation required *de novo* protein synthesis. This is also the case for immediate-early genes such as c-*fos* (Amstad *et al.*, 1992) and c-*myc* (Marcu *et al.*, 1992). Since pradl is a cyclin, we analysed the mRNA expression throughout the cell cycle. Neither in synchronised nor in non-synchronised cells were *pradl* transcripts observed to oscillate (data not shown). Lukas *et al.* (1994) reported that pradl protein synthesis is stringently connected to the mRNA levels, therefore *pradl* expression is mainly under transcriptional control. Addition of actinomycin D (AD) alone had no effect on *pradl* transcript levels (Figure 3b, lane 3).

### *NaB exposure and* plau *expression*

Transcript levels of *plau* were maximally suppressed between 3.0 nd 4.5 mM NaB when analysed after 3 days' treatment (Figure 2a, panel 2). Exposure of N.1 cells to NaB had to exceed 3 h to achieve repression of *plau* mRNA levels (Figure 2b, panel 2, lane 4). Before down-regulation, a slight increase of *plau* transcript levels was observed for reasons which remain obscure (Figure 2b, panel 2, lane 3). Upon CX treatment *plau* mRNA accumulated (not shown).

Fibronectin (*FN*), known to play a positive role during differentiation (Ruoslahti, 1988), was analysed in N.1 cells upon 3.5 mM NaB treatment.

Figure 2b (panel 3) shows that *FN* transcript levels were dramatically increased after 8 h of exposure to the differentiation inducer.

### *NaB exposure and the gene growth arrest-specific 6* (gas6)

NIH 3T3 cells that are growth arrested by serum deprivation start to synthesise *gas6* (Schneider *et al.*, 1988), which is a vitamin K-dependent gene suspected to participate in growth control (Manfioletti *et al.*, 1993). N.1 cells constitutively express *gas6* and the mRNA accumulates when N.1 cells become confluent and retard growth (unpublished observation). In an inverse analogy, when c-*myc* from N.1 cells is induced by mitogens and starts to replicate DNA (unpublished observation), *gas6* levels drop as expected. However, upon a 3 day treatment with 2.0–4.5 mM NaB *gas6* mRNA levels dropped (Figure 3a) rather than increased – although cells arrested growth.

The instability of the *gas6* transcripts involved *de novo* protein synthesis, as shown by CX-induced mRNA accumulation (Figure 3b, lane 2). Exposure of N.1 cells to NaB for only 3 h had no effect on *gas6* expression, (lane 1 *vs* lane 5).

### *Protein kinase A (PKA) dependent signalling*

The distinct kinetics of *pradl* and *plau* down-regulation by NaB (Figure 2b) supported the idea that the mechanisms by which transcriptional repression was achieved might be different. One possibility for alternative gene regulation could be based on the fact that different signal transduction pathways controlled the steady-state expression of the genes under investigation.

Constitutive levels of *pradl* (Figure 4a, lane 1) increased when the activity of cAMP-dependent PKA was blocked by H-89 (lane 3). Just as for *pradl*, constitutive *plau* expression in N.1 cells was clearly co-controlled by PKA, because H-89 treatment resulted in *plau* over-expression (Figure 4a, lane 3).

**Figure 3** (**a**) Response of *gas6* mRNA to increasing doses of NaB. Lane Control: Constitutive expression of untreated N.1 cells. Lanes 0.5–4.5: N.1 cells were exposed for 72 h to 0.5, 2.0, 3.0 and 4.5 mM NaB respectively. (**b**) Constitutive and NaB-modulated *pradl* and *gas6* transcript expression is affected by cyclohexamide and actinomycin D in N.1 cells. CO, Constitutive mRNA expression. NaB, N.1 cells treated with 3.5 mM NaB for 3 h either alone, or in combination with cyclohexamide (CX), actinomycin D (AD) or both (CX + AD). Filters were hybridised simultaneously against *gas6* and *pradl* (upper panels), stripped and rehybridised with a probe against GAPDH (lower panels).

**Figure 4** PKA modulators (**a**) and tumour promoters (**b**) influence constitutive and NaB-suppressed transcript levels of *prad1*, *plau* and *gas6* in N.1 cells. Untreated N.1 cells (−) and N.1 cells that were pretreated with 3.5 mM NaB (+) for 4 h were subsequently exposed (still in the presence of NaB) to the PKA inhibitor H-89, the adenylate cyclase activator forskolin, the PKC activator TPA and thapsigargin, which is a modulator of intracellular $Ca^{2+}$, for another 3 h, or left unexposed (Control). The isolated RNA was hybridised against *prad1* and *gas6* simultaneously (upper panels), stripped, rehybridised against *plau* (middle panels), restripped and rehybridised with a probe against GAPDH (lower panels).

Thus, PKA activity suppressed *prad1*. Accordingly, when PKA was stimulated by forskolin (which is an inducer of adenylate cyclase and therefore increases the cAMP pool, the substrate of PKA) suppression of constitutive *prad1* and *plau* levels were observed (lane 5). When NaB was given in addition to H-89 and forskolin respectively, *prad1* transcript levels were down-regulated without indicating NaB-specific regulation on PKA-dependent signalling. When NaB was given in the presence of forskolin, however, no suppression of *plau* transcripts was observed (lane 6).

### *PKC and $Ca^{2+}$-dependent signalling*

TPA (Figure 4b, lane 3) neither influenced constitutive (lane 1) nor NaB-mediated down-regulation of *prad1* (lane 4). Constitutive *plau* expression (lane 1, panel 2) was dramatically induced by TPA (lane 3). NaB, which down-regulated steady-state *plau* mRNA (lane 2), was entirely ineffective in modulating TPA-mediated overexpression of *plau* transcripts (lane 4).

Thapsigargin induces $Ca^{2+}$ release from intracellular stores (Clapham, 1995). Unexpectedly, thapsigargin slightly decreased, rather than elevated constitutive *prad1* mRNA levels (Figure 4b, lane 5) and co-application of NaB further reduced transcript expression (lane 6).Thus, interference of NaB on *prad1* expression was apparently not specific to early $Ca^{2+}$ signals.

Thapsigargin had no effect on constitutive *plau* mRNA levels (Figure 4b, panel 2, lane 5). NaB administered alone down-regulated *plau* mRNA expression (lane 2). In contrast, co-application of NaB and thapsigargin resulted in *plau* transcript accumulation (lane 6).

The differentiating effect is not terminal: as soon as NaB was removed from the culture medium the transcripts of the investigated genes reappeared and were re-expressed within 12 h.

FN mRNA levels also dropped (Figure 5) when NaB was removed. Thereafter cells changed their morphology to the N.1 phenotype and resumed growing (Krupitza, 1995*b*).

### Discussion

The following conclusions can be drawn from this study. *prad1* is efficiently down-regulated by NaB exposure. The transcript required *de novo* mRNA synthesis for degradation otherwise *prad1* levels should have decreased during inhibition of transcription. This, however, might only be achieved when a *prad1* mRNA controlling gene is synthesised (in terms of both transcription and translation) even earlier than *prad1* itself. Both exposure to CX alone or co-application of CX and AD, resulted in *prad1* mRNA accumulation, suggesting that *prad1* transcript instability required *de novo* translation of another–probably unstable and even earlier–gene for its regulation. Recent work by Daksis *et al.* (1994) identified the c-myc protein as a regulator of the *prad1* oncogene in Rat.1 cells. c-*myc* itself is an immediate-early gene with a very short-lived transcript and protein. Moreover it was found that NaB promotes c-*myc* instability (Herold and Rothberg, 1988; Krupitza *et al.*, 1995*b*), which might finally result in *prad1* down-regulation by NaB.

TPA, which induces c-*myc* (Krupitza *et al.*, 1995*b*) in N.1 cells, failed to induce *prad1*, however, implying a more complex c-*myc*–*prad1* relationship.

In the same way as *prad1*, *plau* was repressed by NaB. Addition of AD, either alone or in combination with CX, kept *plau* mRNA at control levels, whereas CX alone resulted in transcript accumulation (data not shown). Thus, *plau* and *prad1* mRNA degradation by NaB seemed to be controlled by distinct mechanisms.

The data suggested that *gas6* did not exert direct growth control on N.1 cells, because *gas6* was down-regulated in consequence to NaB-mediated growth arrests. In contrast, high *gas6* levels measured in resting cells following serum deprivation (Schneider *et al.*, 1988; G Krupitza, unpublished observation), might indicate the existence of different types of cell cycle arrest conducted by independent genes.

PKA down-regulated constitutive as well as NaB-modulated expression of *prad1* and *plau*. NaB, however, did

**Figure 5** Release from the NaB arrest. N.1 cells were exposed to 3.5 mM NaB for 3 days (Arrest), then the culture medium was discarded, monolayers were rinsed once with prewarmed PBS and were further incubated in the presence of standard medium containing 10% FCS for 2, 4 and 12 h respectively. The filter carrying separated total RNA of N.1 cells was hybridised against *pradl* and *gas6*, stripped, rehybridised against *plau*, restripped and rehybridised against *FN* and GAPDH.

not specifically interact with PKA signalling. Adenylate cyclase contributed to *plau* expression, but not across PKA activation. Since we did not observe *plau* suppression when NaB was applied in the presence of forskolin, maintenance of *plau* transcript levels might be accomplished by a separate signalling pathway that either (co-)controlled *plau* in parallel to the PKA pathway, just enforced by NaB, or which became immediately switched on upon NaB treatment.

PKC activity regulated expression of *plau*, but not that of *pradl*. The experiments using TPA supported the idea that NaB had no direct influence on PKC activity. Previous experiments, however, clearly showed that TPA-mediated c-*myc* induction could be blocked by co-application of NaB (Krupitza *et al*., 1995*b*).

Thus it can be concluded that signals, which are initially generated by, or transduced across PKC, are blocked by NaB somewhere downstream of an unidentified checkpoint, at which signals split up for selected target genes.

$Ca^{2+}$ release from intracellular stores influenced *pradl* expression more in general rather than specifically. *plau*, which was initially not regulated by $Ca^{2+}$-mediated signalling (thapsigargin exposure had no effect on *plau* expression) became sensitive to such signals during NaB treatment (thapsigargin in combination with NaB resulted in transcript accumulation). This could either be achieved by

(i) improved accessibility of *plau* promoter regions (by loosening respective chromatin structures by NaB-mediated histone hyper-acetylation; (Lee *et al*., 1993);

(ii) by inhibition of transcription suppressors; or

(iii) by stimulation of transcription promoters that are capable of co-operating with $Ca^{2+}$-induced signals.

The results described above demonstrate that the effects of even well-characterised bioactive agents on gene expression are unpredictible when applied in combination. Compounds that additively or synergistically repress growth-related genes might oppose repression of genes involved in invasive outgrowth.

There existed a small minority of cells that became multinucleated upon NaB treatment, exhibiting a tremendous increase in plasma mass (giants). Leakiness of NaB on cell cycle specific growth arrest in $G_1$ permits a minority to arrest as late as $G_2$ (Karlsen *et al*., 1991). This fact might have resulted in nuclear division, but not in cytokinesis (Krupitza *et al*., 1995*b*). We have not observed that these cells re-entered the cell cycle. Although these cells cannot be classified as 'terminally differentiated' (in a functional sense), they were probably stably arrested. However, the vast majority of cells was only reversibly blocked (depending on the presence of NaB).

Since NaB induces efficient growth arrest at non-toxic concentrations, the compound might be utilised to study effects on growth factor receptor regulation.

**Acknowledgements**
We thank Dr Rainer DeMartin, Dr Hannes Hofmann and Dr Claudio Schneider for providing probes. This work was supported by the Medizinisch Wissenschaftlicher Fonds des Bürgermeisters der Bundeshauptstadt Wien and by the Theodor Körner Stiftung.

### References

AMSTAD P, KRUPITZA G AND CERUTTI P. (1992). Mechanisms of c-fos induction by active oxygen. *Cancer Res*., **52,** 3952–3960.

ARNOLD A, KIM H, GAZ R, EDDY R, FUKUSHIMA Y, BYERS M, SHOWS T AND KRONENBERG H. (1989). Molecular cloning and chromosomal mapping of DNA rearranged with the parathyroid hormone gene in a parathyroid adenoma. *J. Clin. Invest*., **83,** 2034–2040.

AXELROD J, REICH R AND MISHKIN R. (1989). Expression of human recombinant plasminogen activator enhances invasion and experimental metastasis of H-ras transformed NIH-3T3 cells. *Mol. Cell. Biol*., **9,** 2133–2141.

BLOCH A. (1984). Induced cell differentiation in cancer therapy. *Cancer Treat. Rep*., **68,** 199–205.

BUCKLEY M, SWEENEY K, HAMILTON J, SINI R, MANNING D, NICHOLSON R, deFAZIO J, WATTS C, MUSGROVE E AND SUTHERLAND R. (1993). Expression and amplification of cyclin genes in human breast cancer. *Oncogene*, **8,** 2127–2133.

BUICK R, PULLANO R AND TRENT J. (1985). Comparative properties of five human ovarian adenocarcinoma cell lines. *Cancer Res*., **45,** 3668–3676.

CASTAIGNE S, CHOMIENNE C, DANIEL M, BALLERINI P, BERGER R, FENAUX P AND DEGOS L. (1990). All-trans retinoic acid as a differentiation therapy for acute promyelocytic leukemia. I. clinical results. *Blood*, **76,** 1704–1709.

CLAPHAM D. (1995). Calcium signaling. *Cell*, **80,** 259–268.

DAKSIS J, LU R, FACCHINI L, MARHIN W AND PENN L. (1994). Myc induces cyclin D1 expression in the absence of *de novo* protein synthesis and links mitogen-stimulated signal transduction to the cell cycle. *Oncogene*, **9,** 3635–3645.

DOWDY S, HINDS P, LOUIE K, REED S, ARNOLD A AND WEINBERG R. (1993). Physical interaction of the retinoblastoma protein with human D cyclins. *Cell*, **73,** 499–511.

FISCHKOFF S, HOESSLY M AND ROSSI R. (1990). Characterization of sublines of HL-60 human leukemia cells resistant to induction of differentiation by butyric acid. *Leukemia*, **4,** 302–306.

GRUNT T, DITTRICH E, SOMAY C, WAGNER T AND DITTRICH C. (1991). Separation of clonogenic and differentiated cell phenotypes of ovarian cancer cells (HOC-7) by discontinuous density gradient centrifugation. *Cancer Lett*., **58,** 7–16.

HAGUE A, MANNING A, HANLOW K, HUSCHTSCHA L, HART D AND PARASKEVA C. (1993). Sodium butyrate induces apoptosis in human colonic tumor cell lines in a p53-independent pathway: implications for the possible role of dietary fibre in the prevention of large bowel cancer. *Int. J. Cancer*, **55,** 498–505.

HEROLD K AND ROTHBERG P. (1988). Evidence for a labile intermediate in the butyrate induced reduction of the level of c-myc RNA in SW837 rectal carcinoma cells. *Oncogene*, **3,** 423–428.

JIANG W, KAHN S, ZHOU P, ZHANG Y, CACACE A, INFANTE A, DOI S, SANTELLA R AND WEINSTEIN B. (1993). Overexpression of cyclin D1 in rat fibroblasts causes abnormalities in growth control, cell cycle progression and gene expression. *Oncogene*, **8**, 3447–3457.

KARLSEN AE, FUJIMOTO WY, RABINOVITCH P, DUBE S AND LERNMARK A. (1991). Effects of sodium butyrate on proliferation dependent insulin gene expression and insulin release in glucose sensitive RIN-5AH cells. *J. Biol. Chem.*, **266**, 7542–7548.

KRUPITZA G, HULLA W, HARANT H, DITTRICH E, KALLAY E, HUBER H, GRUNT T AND DITTRICH C. (1995*a*). Retinoic acid induced death of ovarian carcinoma cells correlates with c-*myc* stimulation. *Int. J. Cancer*, **61**, 649–657.

KRUPITZA G, HARANT H, DITTRICH E, SZEKERES T, HUBER H AND DITTRICH C. (1995*b*). Sodium butyrate inhibits c-*myc* splicing and interferes with signal transduction in ovarian carcinoma cells. *Carcinogenesis*, **16**, 1199–1205.

KRUPITZA G, FRITSCHE R, DITTRICH E, HARANT H, HUBER H, GRUNT T AND DITTRICH C. (1995*c*). Macrophage colony-stimulating factor is expressed by an ovarian carcinoma subline and stimulates the c-*myc* proto-oncogene. *Br. J. Cancer*, **72**, 35–40.

LEE D, HAYES J, PRUSS D AND WOLFFE A. (1993). A positive role for histone acetylation in transcription factor access to nucleosomal DNA. *Cell*, **72**, 73–84.

LIOTTA L, STEEG P AND STETLER-STEVENSON W. (1991). Cancer metastasis and angiogenesis: An imbalance of positive and negative regulation. *Cell*, **64**, 327–336.

LIPPMAN S, PARKINSON D, ITRI L, WEBER R, SCHANTZ S, OTA D, SCHUSTERMAN M, KRAKOFF I, GUTTERMAN J AND HONG W. (1992). 13-cis retinoic acid and interferon alpha-2a: Effective combination therapy for advanced squamous cell carcinoma of the skin. *J. Natl Cancer Inst.*, **84**, 235–241.

LUKAS J, PAGANO M, STASKOVA Z, DRAETTA G AND BARTEK J. (1994). Cyclin D1 protein oscillates and is essential for cell cycle progression in human tumor cell lines. *Oncogene*, **9**, 707–718.

MANFIOLETTI G, BRANCOLINI C, AVANZI G AND SCHNEIDER C. (1993). The protein encoded by a growth arrest specific gene (gas6) is a new member of the vitamin K-dependent proteins related to protein S, a negative coregulator in the blood coagulation cascade. *Mol. Cell. Biol.*, **13**, 4976–4985.

MARCU K, BOSSONE S AND PATEL A. (1992). *myc* function and regulation. *Annu. Rev. Biochem.*, **61**, 809–860.

MONTGOMERY A, DeCLERCK Y, LANGLEY K, REISFELD R AND MUELLER B. (1993). Melanoma-mediated dissolution of extracellular matrix: contribution of urokinase-dependent and metalloproteinase-dependent proteolytic pathways. *Cancer Res.*, **53**, 693–700.

NOVOGRODSKY A, DVIR A, RAVID A, SHKOLNIK T, STENZEL K, RUBIN A AND ZAIZOV R. (1983). Effect of polar organic compounds on leukemic cells: Butyrate induced partial remission of acute myelogenous leukemia in a child. *Cancer*, **51**, 9–14.

RABBITTS, TH. (1991). Translocations, master genes, and differences between the origins of acute and chronic leukemias. *Cell*, **67**, 641–644.

RUOSLAHTI E. (1988). Fibronectin and its receptors. *Annu. Rev. Biochem.*, **57**, 375–413.

SCHNEIDER C, KING R AND PHILIPSON L. (1988). Genes specifically expressed at growth arrest of mammalian cells. *Cell*, **54**, 787–793.

STAIANO-COICO L AND HIGGINS P. (1992). Cell shape changes during transition of basal keratinocytes to mature enucleate-cornified envelopes: Modulation of terminal differentiation by fibronectin. *Exp. Cell Res.*, **201**, 126–136.